# Camera-Radar Fusion with Radar Channel Extension and Dual-CBAM-FPN for Object Detection

**DOI:** 10.3390/s24165317

**Published:** 2024-08-16

**Authors:** Xiyan Sun, Yaoyu Jiang, Hongmei Qin, Jingjing Li, Yuanfa Ji

**Affiliations:** 1 Guangxi Key Laboratory of Precision Navigation Technology and Application, Guilin University of Electronic Technology, Guilin 541004, China; sunxiyan@guet.edu.cn (X.S.); 22022201045@mails.guet.edu.cn (Y.J.); 21022303138@mails.guet.edu.cn (H.Q.); 2 Information and Communication School, Guilin University of Electronic Technology, Guilin 541004, China; 3 National & Local Joint Engineering Research Center of Satellite Navigation Positioning and Location Service, Guilin 541004, China; 4 GUET-Nanning E-Tech Research Institute Co., Ltd., Nanning 530031, China

**Keywords:** object detection, camera-radar fusion, FPN, radar channel extension, CBAM

## Abstract

When it comes to road environment perception, millimeter-wave radar with a camera facilitates more reliable detection than a single sensor. However, the limited utilization of radar features and insufficient extraction of important features remain pertinent issues, especially with regard to the detection of small and occluded objects. To address these concerns, we propose a camera-radar fusion with radar channel extension and a dual-CBAM-FPN (CRFRD), which incorporates a radar channel extension (RCE) module and a dual-CBAM-FPN (DCF) module into the camera-radar fusion net (CRF-Net). In the RCE module, we design an azimuth-weighted RCS parameter and extend three radar channels, which leverage the secondary redundant information to achieve richer feature representation. In the DCF module, we present the dual-CBAM-FPN, which enables the model to focus on important features by inserting CBAM at the input and the fusion process of FPN simultaneously. Comparative experiments conducted on the NuScenes dataset and real data demonstrate the superior performance of the CRFRD compared to CRF-Net, as its weighted mean average precision (wmAP) increases from 43.89% to 45.03%. Furthermore, ablation studies verify the indispensability of the RCE and DCF modules and the effectiveness of azimuth-weighted RCS.

## 1. Introduction

Road traffic safety and efficiency are the key challenges in modern transportation [1]. An intelligent roadside unit (RSU), which is capable of achieving over-the-horizon perception, is used to monitor traffic flow and alert road users to dangerous situations [2,3]. To achieve this, intelligent RSUs must possess a stable and reliable perception system [1].

However, due to the complexities presented by the road environment, such as object occlusion, rain, and lighting changes, a single sensor cannot meet the demand for reliable environment perception; this has prompted the emergence of multi-sensor fusion as a research hotspot [4]. Among the variety of sensors utilized in intelligent transportation, millimeter-wave radar and cameras exhibit complementary characteristics. Specifically, millimeter-wave radars are robust to severe weather conditions, providing accurate velocity and depth information of objects. However, their azimuth resolution is limited, and they fail to recognize the appearance and shape of objects. Cameras, on the other hand, are capable of achieving a high azimuth resolution, accurately capturing information about objects’ physical characteristics; however, they fall short when it comes to depth information and are vulnerable to weather conditions and lighting conditions. Therefore, the fusion of the millimeter-wave radar and a camera has attracted significant attention from researchers [5].

Compared to single sensors, which have issues with misidentification or, in some cases, failure to detect objects altogether, multi-sensor fusion is an advanced object detection method. Several researchers have used it to great effect; J. Dudczyk et al. [6] realized object detection through a multi-sensory data fusion method, while X. Liu et al. [7] iteratively updated the radar and camera through an interactive module and aggregated the radar and image features with a set of sparse 3D object queries, all while preserving the integrity of the original radar features to prevent information loss to facilitate more accurate object detection. In another experiment, H. Sun et al. [8] used height extension and azimuth uncertainty to achieve more significant feature extraction, generating denser radar input and achieving better alignment between the radar data and the objects on the image. M. Zong et al. [9] designed a spatial attention concatenate fusion (SAC) module that focuses on the feature layer, combining the positional information of shallow feature maps with deep semantic information, to improve the detection of both large and small targets. Meanwhile, S. He et al. [10] created the multi-scale fusion obstacle detection algorithm, which combines different scales of radar and visual feature maps at three different levels, making it easier for the model to detect small targets in the environment.

Radar and camera fusion methods can be classified as data-level, feature-level, and decision-level fusion based on the fusion stage. Most studies fusing the mmWave radar data and camera data of intelligent RSUs take place at the decision level. J. Lin et al. [3] proposed a deep-learning-based early fusion between a mmWave radar and an RGB camera sensor, but the feature extraction prior to fusion was too simple. Since radar data and camera data have distinct representations, feature-level fusion emerged as the more suitable approach. The feature-level fusion of radar data and camera data currently encompasses two approaches: fusion after feature extraction and fusion during feature extraction. For fusion that takes place after feature extraction, radar features and camera features extracted from the last layer of each extraction network are employed [11,12,13]. This method ignores the low-level features, and as such, its capacity to detect smaller objects is limited. When the fusion takes place during feature extraction however, radar features and camera features extracted from each layer of each extraction network are all used to good advantage [14,15,16]. By utilizing the high-level and low-level features simultaneously, fusion that takes place during feature extraction considers both small and larger objects. Camera-radar fusion net (CRF-Net) is a representative model of fusion during feature extraction, which builds on RetinaNet with a Visual Geometry Group-16 (VGG16) backbone and a feature pyramid network (FPN) [17]. Radar and camera data are concatenated and fed into the VGG16 network; then, the radar data are concatenated to the output of the previous fused network block. Subsequently, the FPN is introduced to enhance the fusion of the radar features. CRF-Net automatically learns the level at which the fusion of the sensor data is most beneficial for the detection result, making it a highly promising method [18].

However, CRF-Net has several issues that have yet to be resolved. First, it uses limited radar channels, which are generated by distance and radar cross-section (RCS). Second, within the FPN structure, there is a lack of emphasis on important features. This inevitably affects the feature extraction of objects whose features are less prominent, such as small and occluded objects, and may have a negative effect on the detection performance. Therefore, it is crucial to minimize information loss during feature representation [19] and enhance the representation ability of important features during feature fusion [20] to improve the performance of CRF-Net.

To solve the abovementioned problems, we propose camera-radar fusion with radar channel extension and dual-CBAM-FPN (CRFRD), a novel camera-radar fusion model based on CRF-Net for object detection. By extending three radar channels and inserting CBAM at the input and the fusion process of FPN simultaneously, the CRFRD effectively enhances object detection accuracy.

The main contributions of this paper are as follows:

- We propose the CRFRD model, which incorporates radar channel extension (RCE) and dual-CBAM-FPN (DCF) into the CRF-Net model. The CRFRD enriches the representation of radar features and pays more attention to the important information of multi-scale features that are fused by radar and camera features.
- We introduce a new parameter azimuth-weighted RCS to construct a radar channel, making use of the azimuth and RCS parameters to achieve richer feature representation. Along with velocity, azimuth and azimuth-weighted RCS are chosen to construct additional radar channels and undergo experimental evaluation.
- We present a dual-CBAM-FPN strategy to direct the model’s focus toward pivotal features along the channel and spatial dimensions. CBAM is inserted into both the input and the fusion process of FPN, which significantly enhances the feature representation, particularly with regard to smaller objects.
- Numerous experiments verify the effectiveness of the CRFRD model in improving the detection accuracy of CRF-Net. The weighted mean average precision (wmAP) increases from 43.89% to 45.03%, and more small and occluded objects are detected by CRFRD.

The remainder of the paper is structured as follows. In Section 2, we introduce a series of relevant studies and provide a concise overview of our background knowledge in this area. In Section 3, we describe the structure of the proposed model, as well as that of RCE and DCF. In Section 4, we present the results and analysis of comparative experiments and ablation studies. Finally, in Section 5, we list the conclusions of our work.

## 2. Related Work and Background Knowledge

### 2.1. Related Work

The limited utilization of radar features and insufficient extraction of important features are not unique to CRF-Net, and numerous researchers have endeavored to address or mitigate these problems.

Two common approaches are used to fuse the different representations of radar data and camera data. One involves projecting the radar point clouds onto the image plane to construct additional channels with parameters of the radar point clouds [19], from which 2D bounding boxes are obtained. Another option is to project the multi-view camera features to the 3D ego-car coordinate to generate bird’s eye view (BEV) features [21], which provide 3D bounding boxes. In this paper, we focused on the fusion of RSUs, making multi-view camera features more difficult to achieve; therefore, we chose to project the radar point clouds onto the image plane to generate 2D bounding boxes for object detection. According to NuScenes, a comprehensive dataset for radar-camera fusion, there are 18 parameters for each radar point, including the position coordinate in three dimensions (x,y,z), the radial velocity in the x and y directions $\left(v_{x},v_{y}\right)$, dynamic properties $p_{D}$ indicating whether the point is stationary or in motion, point identification, RCS σ, radial velocity compensated by ego-motion in the x and y directions $\left(v_{x\_comp},v_{y\_comp}\right)$, clustering validity states, Doppler ambiguity solution states, the false-alarm probability of the cluster, quality validity states, and the uncertainty of x and y position and velocity $\left(x_{rms},y_{rms},v_{x_{rms}},v_{y_{rms}}\right)$ [22]. In most cases, distance d and azimuth θ are also calculated to assist with the construction of radar channels. The parameters chosen for individual models may vary. For instance, S. Chang et al. [23] created the radar channels using three parameters, including distance and velocity $\left(d,v_{x},v_{y}\right)$. These three parameters were also implemented by several other researchers [11,24]. L. Li et al. [22] selected five parameters to construct the radar channels, including RCS, distance, velocity, and azimuth $\left(\sigma ,d,v_{x},v_{y},\theta \right)$. In addition to several of the previously mentioned parameters, L. Stacker et al. [19] proposed two novel parameters which they referred to as azimuth uncertainty and azimuth-uncertainty-weighted RCS (UwRCS), respectively, and constructed the radar channels using one of the proposed parameters together with the distance d and RCS σ. They then compared the model’s performance with different proposed parameters, demonstrating that a model using UwRCS outperformed the model that implemented azimuth uncertainty. Constructing more comprehensive radar channels, either by leveraging more radar output parameters or through the design of new representation parameters, to minimize the loss of radar feature information is an area that merits further investigation.

FPN is a network structure that extracts features from different layers and makes full use of multi-scale features. However, it is susceptible to loss and underutilization of information during the multi-scale fusion process. Introducing the attention mechanism has proven to be an effective solution to the problem [25]. The convolutional block attention module (CBAM), an effective mechanism for enhancing convolutional neural networks (CNNs), has been inserted into the FPN model to improve its performance. The combination of CBAM and FPN takes three distinctive forms. First, CBAM is incorporated in the backbone network [26], where it takes the information refined by the spatial and channel attention mechanism as the input of FPN $C_{i}$. This method emphasizes the local efficient information of the feature maps, enhancing the detection capability for occluded or truncated objects. Second, CBAM is inserted into the input or output of FPN [27,28]. Q. Guo et al. [27] added CBAM before the FPN output of the lowest-level feature map to emphasize the object region of interest, improving the model’s ability to detect small objects. J.C.Á. Cerón et al. [28] employed CBAM before each feature map $C_{i}$ input into FPN and before FPN output each feature map $P_{i}$, respectively. This aggregation of local information with its corresponding global dependencies extracts richer context, refining the feature representation of objects. Third, CBAM is introduced into the fusion process of each scale of the feature maps [29,30]. In their work, Z. Gui et al. [29] began by upsampling the high-level features of FPN $P_{i}$ and inputting them into CBAM to obtain the attention-weighted features $A_{i-1}$, which were further fused with $C_{i-1}$ via concatenation to receive the refined features $P_{i-1}$. These refined feature maps highlight salient features at specific scales. Y. Han et al. [30] used the upsampled high-level features of FPN and $C_{i-1}$ as the input of CBAM to obtain the refined feature maps $P_{i-1}$. By improving the feature representation, this model extracts important features to enhance detection accuracy. All the above-described combinations of CBAM with FPN emphasize the local information of the feature map. When inserting CBAM at the input or output of FPN, the feature map in each scale only focuses on its own local information. When CBAM is inserted during the fusion of feature maps at each scale, low-level feature maps focus on the local information of feature maps fused by higher-level feature maps. It is worth fully leveraging the various forms of CBAM-FPN to enhance the multi-scale feature fusion performance of FPN.

### 2.2. Radar Data Preprocessing

To generate radar images, it is necessary to solve the problems of projection to the image plane, height loss, and data sparsity. The radar data are processed via spatial calibration, line expansion, and aggregation of multiple radar sweeps.

**Figure 1:**
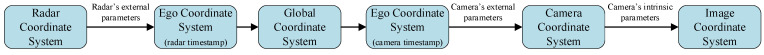
Flow diagram of coordinate transformation from radar coordinate system to image coordinate system. The radar points in the radar coordinate system are rotated and translated to the ego coordinate system under the radar timestamp using the radar’s external parameters then converted to the global coordinate system. After that, the global coordinates are transformed to the ego coordinates under the camera timestamp then transformed into camera coordinates using the camera’s external parameters. Finally, the camera coordinates are transformed into image coordinates using the camera’s intrinsic parameters.

Radar and camera data are composed of two different structures, with different coordinate systems. In order to fuse radar data with camera data, it is essential to unify the coordinates of the two sensors. This is known as spatial calibration—a coordinate transformation between the radar coordinate system, the global coordinate system, the camera coordinate system, and the image coordinate system, which allows the radar point clouds to be projected onto the image plane [15]. The coordinate transformation process is depicted in Figure 1. First, the radar points in the radar coordinate system are rotated and translated to the ego coordinate system under the radar timestamp using the radar’s external parameters. Then, they are converted to the global coordinate system. After that, the global coordinates are transformed to the ego coordinates under the camera timestamp. Next, the ego coordinates under the camera timestamp are transformed to camera coordinates using the camera’s external parameters. Finally, the camera coordinates are transformed to the image coordinates using the camera’s intrinsic parameters. When the platform installing the radar and camera is moving, the ego coordinate system under the radar timestamp may differ from the ego coordinates under the camera timestamp; when the platform is stationary, the ego coordinate system under the radar timestamp, the global coordinate system and the ego coordinates under the camera timestamp are identical. To generate the radar image, the parameters of the radar point clouds are stored as pixel values for positions in the image plane; these should be converted to a value between 0 and 255. At the location of image pixels where no radar returns are present, the pixel values are filled by 0. The radar image plane generated by each radar parameter serves as a channel for the radar image [22].

**Figure 2:**
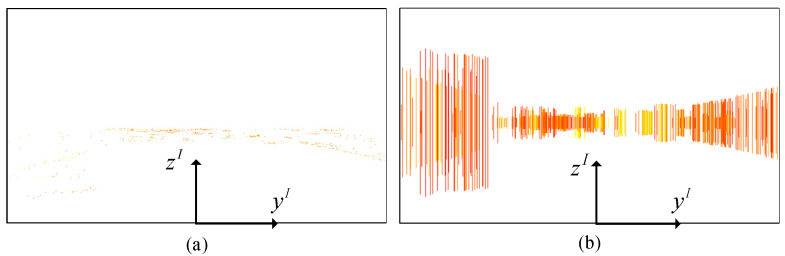
Projection results of radar point clouds onto the image plane: (**a**) is the projection result prior to the line expansion, whereas (**b**) is the projection result after the line expansion. The image coordinate system was used here and is denoted as $\left(y^{I},z^{I}\right)$.

As radar detection does not provide information about objects’ height, the radar point projection is extended along the vertical direction to three meters [19]. The radar point is mapped into the image plane with a pixel width of one. Figure 2 shows the projection results of radar point clouds onto the image plane, where Figure 2a is the projection result prior to the line expansion and Figure 2b is a projection result after the line expansion. Figure 2 depicts the image coordinate system, which is denoted as $\left(y^{I},z^{I}\right)$.

Radar sensors can only provide limited point clouds for object detection and are therefore incapable of performing classification. To address the sparsity of radar point clouds, we accumulate the point clouds data from several radar scans as a single frame. This significantly enhances the density of the data [17].

### 2.3. CRF-Net

CRF-Net takes the camera image $I^{camera}\in R^{m\times n\times l_{c}}$ and the generated radar image $I^{radar}\in R^{m\times n\times l_{r}}$ as inputs, where m and n represent the dimensions of the images; $l_{c}$ represents the number of channels of the camera image, $l_{c}=3$; and $l_{r}$ represents the number of channels of the radar image, $l_{r}=2$. The camera image and the radar image are fused by concatenation and, thereafter, are considered as the input of the VGG16 network, namely, $C_{0}=I^{radar}⊕I^{camera}$, where ⊕ represents concatenation. Subsequently, the output of each block of the VGG16 network undergoes further concatenation with the max-pooled radar images $F_{i}^{radar}$ to obtain the fused features $C_{i}$.

(1) $$\{\begin{array}{c} F_{i}^{radar}=MaxPool\left(F_{i-1}^{radar}\right),\\ C_{i}=Block_{i}\left(C_{i-1}\right)⊕F_{i}^{radar}, \end{array}\begin{array}{ccc} & & i=1,2,\cdots ,5 \end{array},$$

where $F_{0}^{radar}=I^{radar}$. MaxPool(⋅) represents maximum pooling and $Block_{i}\left(\cdot \right)$ represents the output after the i-th block of VGG16.

During feature extraction of VGG16 and multi-scale feature fusion of FPN, the radar channels undergo additional concatenation at each level. As shown in Equations (2) and (3), using $C_{3}∼C_{5}$ and $F_{3}^{radar}∼F_{7}^{radar}$ as the input, FPN outputs five feature maps $N_{3}∼N_{7}$, which are in different scales [31].

(2) $$P_{i}=\{\begin{array}{c} {\mathrm{Conv}}_{3\times 3}\left({\mathrm{Conv}}_{1\times 1}\left(C_{i}\right)+\mathrm{Upsample}\left({\mathrm{Conv}}_{1\times 1}\left(C_{i+1}\right)\right)\right),\\ {\;\mathrm{Conv}}_{3\times 3}\left({\mathrm{Conv}}_{1\times 1}\left(C_{i}\right)\right),\;\\ {\mathrm{Conv}}_{3\times 3}\left(C_{i-1}\right),\\ {\mathrm{Conv}}_{3\times 3}\left(\mathrm{Relu}\left(P_{i-1}\right)\right), \end{array}\begin{array}{c} i=3,4\\ i=5\\ i=6\\ i=7 \end{array},$$

(3) $$N_{i}=F_{i}^{radar}⊕P_{i},i=3,4,\cdots ,7$$

where $P_{i}$ represents the intermediate feature maps of FPN; ${\mathrm{Conv}}_{1\times 1}\left(\cdot \right)$ represents 2D convolution with 1 × 1 convolution kernel and stride 1, which is used to convert the number of channels of the feature map; ${\mathrm{Conv}}_{3\times 3}\left(\cdot \right)$ represents 2D convolution with a 3 × 3 convolution kernel; and Upsample(⋅) represents upsampling. The purpose of the upsampling operation is to obtain a dimension that can be added to the previous layer; Relu(x)=max(0,x).

The loss function of CRF-Net consists of a classification loss $L_{cls}$ and a regression loss $L_{reg}$. The total training loss $L_{total}$ for the whole task is constructed as Equation (4).

(4) $$L_{total}\left(x_{i},y_{i}\right)=L_{cls}\left(x_{i},{x^{\prime }}_{i}\right)+\beta L_{reg}\left(y_{i},{y^{\prime }}_{i}\right)$$

(5) $$L_{cls}\left(x_{i},{x^{\prime }}_{i}\right)=\{\begin{array}{cc} -\alpha {\left(1-{x^{\prime }}_{i}\right)}^{\gamma }\log\left({x^{\prime }}_{i}\right),\; & x_{i}=1\\ -\left(1-\alpha \right){\left({x^{\prime }}_{i}\right)}^{\gamma }\log\left(1-{x^{\prime }}_{i}\right), & x_{i}=0 \end{array}$$

(6) $$L_{reg}\left(y_{i},{y^{\prime }}_{i}\right)=\{\begin{array}{cc} 0.5\delta^{2}{\left(y_{i}-{y^{\prime }}_{i}\right)}^{2},\; & \begin{array}{cc} if & \left|y_{i}-{y^{\prime }}_{i}\right|\leq \frac{1}{\delta^{2}} \end{array}\\ \left|y_{i}-{y^{\prime }}_{i}\right|-\frac{0.5}{\delta^{2}}, & \mathrm{otherwise} \end{array}$$

where i is the anchor index; $x_{i}$ is the ground truth score, which is 1 if the anchor is positive and 0 if it is negative; ${x^{\prime }}_{i}$ is the model’s estimated probability for the class with the label $x_{i}=1$; $y_{i}$ is the ground truth of the bounding box; ${y^{\prime }}_{i}$ is the predicted output of the regression subnetwork; and β denotes the weight of $L_{reg}$. The classification loss $L_{cls}$ is computed as described by the authors of [32], where α=0.25, γ=2.0. The regression loss $L_{reg}$ is computed as stated by the authors of [33], where δ=2.0, β=1.0.

### 2.4. CBAM

**Figure 3:**
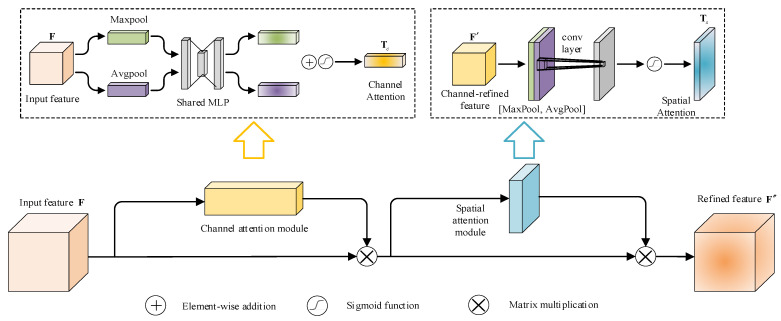
CBAM. The input feature is processed by the channel attention module and the spatial attention module in turn to obtain a refined feature. The channel attention module utilizes both max-pooling outputs and average-pooling outputs with a shared network; the spatial attention module utilizes similar two outputs that are pooled along the channel axis and forwarded to a convolution layer.

CBAM is a lightweight and general attention module that combines the channel attention mechanism and spatial attention mechanism for feed-forward convolutional neural networks. It can conduct end-to-end training with basic CNN and achieves a guaranteed performance improvement with less overhead [34]. The channel attention module enhances the feature representation of different channels, while spatial attention facilitates the extraction of important information regarding various locations in the space [35]. The structure of CBAM is illustrated in Figure 3. The input feature map $F\in R^{H\times W\times C}$ is processed by the channel attention weights $T_{c}\in R^{1\times 1\times C}$ and the spatial attention weights $T_{s}\in R^{H\times W\times 1}$ in turn, which allows the refined feature map $F^{\prime \prime }\in R^{H\times W\times C}$ to be obtained.

(7) $$\begin{array}{l} F^{\prime }=T_{c}⊗F\\ F^{\prime \prime }=T_{s}⊗F^{\prime } \end{array}$$

#### 2.4.1. Channel Attention Module

The channel attention module focuses on the specific meaningful contents of input feature maps [36]. The spatial information of a feature map is aggregated using both average pooling and max-pooling operations, and two different spatial context descriptors are generated. These descriptors are then fed into a shared network composed of a multi-layer perceptron (MLP) to learn the importance of each channel adaptively. The output feature vectors are summed and then activated by a sigmoid function to generate the channel attention weights $T_{c}\in R^{1\times 1\times C}$, which are calculated as

(8) $$T_{c}\left(F\right)=Sigmoid\left(MLP\left(AvgPool\left(F\right)\right)+MLP\left(MaxPool\left(F\right)\right)\right).$$

#### 2.4.2. Spatial Attention Module

The spatial attention module places greater emphasis on the specific locations of input feature maps [37]. Initially, the max-pooling and average-pooling operations are applied along the channel axis to generate features with different context scales. Then, the outcomes of both operations are concatenated along the channel axis to create a feature map with different scales of contextual information. Finally, a 2D convolution with 7 × 7 convolution kernel is utilized to generate a spatial attention weighted map, which is normalized with the sigmoid function $T_{s}\in R^{H\times W\times 1}$. The calculation formula is as follows:

(9) $$T_{s}\left(F^{\prime }\right)=Sigmoid\left({\mathrm{Conv}}_{7\times 7}\left(\left[AvgPool\left(F^{\prime }\right);\;MaxPool\left(F^{\prime }\right)\right]\right)\right).$$

## 3. Camera-Radar Fusion with RCE and DCF

### 3.1. The Overall Structure of CRFRD

**Figure 4:**
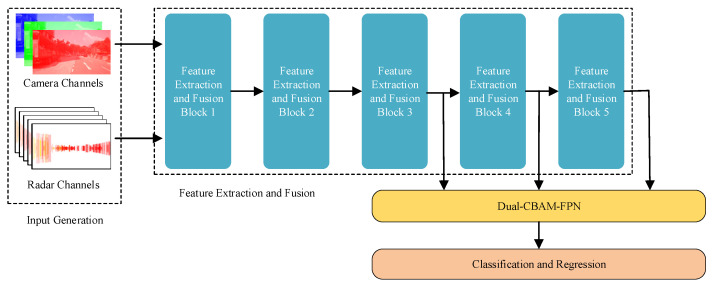
The block diagram of CRFRD is composed of four modules: input generation, feature extraction and fusion, dual-CBAM-FPN, and classification and regression. Input generation is a generator module for camera and radar channels. The feature extraction and fusion module consists of five feature extraction and fusion blocks, with max-pooling and VGG16 being the most important. Dual-CBAM-FPN combines CBAM and FPN. The classification and regression module fulfills the task of object detection.

The block diagram of the CRFRD, shown in Figure 4, is divided into four main components: the input channel generation module, the feature extraction and fusion module, the dual-CBAM-FPN module, and the classification and regression module. The input channel generation module includes camera channel and radar channel generation. Camera images are composed of R, B, and G channels, while radar images comprise five channels: two that are the same as the baseline and three additional channels. The feature extraction and fusion module fuses the camera and radar features during the feature extraction process, since it is based on VGG16, and this means that the features extracted from each block are all fused features. For the dual-CBAM-FPN module, CBAM is inserted into the input and the fusion process of FPN simultaneously. Meanwhile, for the classification and regression module, the fused features are used for both classification and regression tasks.

### 3.2. Radar Channel Extension

In this section, we describe the radar channel extension module of CRFRD, adding three channels to enrich the representations of radar features.

The radial velocity in the x and y directions $\left(v_{x},v_{y}\right)$ indicate the motion state of point clouds. Point clouds with the same radial velocity in the x and y directions are likely to be part of the same motion entity. This facilitates the clustering of point clouds and helps with the identification of different objects, especially those that are occluded. Therefore, the two parameters $\left(v_{x},v_{y}\right)$ are utilized for image plane projection, and two additional radar images are constructed; there are then considered to be two extra radar channels.

Acknowledging the inherent redundancy of azimuth with respect to the position and distance of point clouds, we introduce a new parameter named azimuth-weighted RCS, rather than directly projecting it to the image plane to construct radar image. In the Experimental Section, we evaluate how introducing either the azimuth or the azimuth-weighted RCS as a channel affects the detection accuracy.

According to the description of spatial calibration in Section 2.2, the i-th radar point is projected from the 3D coordinates in the radar coordinate system $P_{i}^{R}=\left(x_{i}^{R},y_{i}^{R},z_{i}^{R}\right)$ to the 2D coordinates in the image coordinate system $P_{i}^{I}=\left(y_{i}^{I},z_{i}^{I}\right)$ through a series of matrix transformations.

(10) $$\begin{array}{l} \left(y_{i}^{I},z_{i}^{I}\right)=T_{C}^{I}\left(x_{i}^{C},y_{i}^{C},z_{i}^{C}\right)\\ =T_{C}^{I}T_{E(t)}^{C}T_{G}^{E(t)}T_{E(t-k)}^{G}T_{R}^{E(t-k)}\left(x_{i}^{R},y_{i}^{R},z_{i}^{R}\right) \end{array},$$

where R, C, and I represent the radar, camera, and image coordinate system, respectively; $T_{Y}^{X}$ represents the transformation from a Y coordinate system to an X coordinate system; E represents the vehicle coordinate system; t represents time; k represents the delay between the radar timestamp and the camera timestamp; and G represents the global coordinate system. The conversion from $\left(x_{i}^{R},y_{i}^{R},z_{i}^{R}\right)$ to $\left(x_{i}^{C},y_{i}^{C},z_{i}^{C}\right)$ is achieved by a rotation matrix and translation vector from 3D to 3D without any loss of information. However, the projection to the image plane loses one dimension of information when converted from the camera coordinate system to the image coordinate system.

(11) $$\{\begin{array}{c} y_{i}^{I}=f\frac{y_{i}^{C}}{x_{i}^{C}}\\ z_{i}^{I}=f\frac{z_{i}^{C}}{x_{i}^{C}} \end{array},$$

where f is the distance between the origin of the image coordinate system and the origin of the camera coordinate system. The information loss can be compensated by $d_{i}^{C}=\sqrt{{\left(x_{i}^{C}\right)}^{2}+{\left(y_{i}^{C}\right)}^{2}+{\left(z_{i}^{C}\right)}^{2}}$ or $\theta_{i}^{C}=\arctan\left(y_{i}^{C}/x_{i}^{C}\right)$ or $\varphi_{i}^{C}=\arctan\left(\sqrt{{\left(x_{i}^{C}\right)}^{2}+{\left(y_{i}^{C}\right)}^{2}}/z_{i}^{C}\right)$. Assuming that $d_{i}^{C}$ is used for compensation, both $\theta_{i}^{C}$ and $\varphi_{i}^{C}$ are functions of $d_{i}^{C}$, $y_{i}^{I}$, and $z_{i}^{I}$. Since $\left(d_{i}^{C},\theta_{i}^{C},\varphi_{i}^{C}\right)$ and $\left(d_{i}^{R},\theta_{i}^{R},\varphi_{i}^{R}\right)$ can be converted from each other, $\theta_{i}^{R}$ is also a function of $d_{i}^{R}$, $y_{i}^{I}$, and $z_{i}^{I}$, which can be denoted as

(12) $$\theta_{i}^{R}=H\left(d_{i}^{R},y_{i}^{I},z_{i}^{I}\right).$$

Here, only the relationship between $\theta_{i}^{R}$ and $d_{i}^{R}$ is discussed. The relationship between $\varphi_{i}^{R}$ and $d_{i}^{R}$ is neglected because in the radar coordinate system, $z_{i}^{R}=0$ and $\varphi_{i}^{R}=90^{\circ }$.

According to Equation (12), if parameter $\theta_{i}^{R}$ is utilized to construct a radar image, it can be considered as a redundancy of a radar image constructed from distance, and it only increases the weight of the distance channel. Therefore, in this paper, we utilize $\theta_{i}^{R}$ in a novel way to construct a new parameter that will enhance the RCS of the targets whose azimuth is close to zero radian.

Azimuth-weighted RCS $\sigma_{\theta }$: We design $\sigma_{\theta }$ by multiplying the azimuth and RCS as follows:

(13) $$\sigma_{\theta }=\theta_{i}^{R}\cdot \sigma .$$

**Figure 5:**
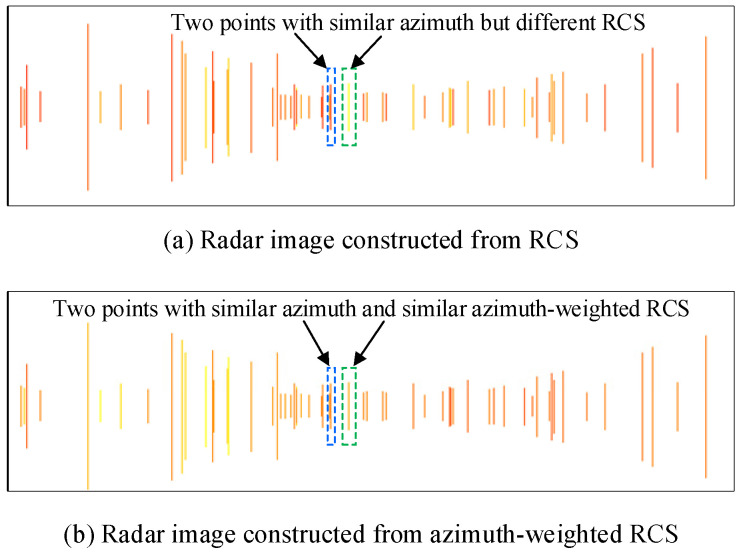
Comparison of radar image constructed from RCS and azimuth-weighted RCS. The blue dashed and green dashed rectangles indicate two different radar points: (**a**) shows two points with similar azimuths but different RCS; (**b**) shows that the points with similar azimuths also have similar azimuth-weighted RCS, indicating that the RCS of the point represented by the green dashed rectangle is enhanced after azimuth weighting.

For two points with different RCS but similar azimuths close to zero radian, $\sigma_{\theta }$ for these two points are both close to zero. After normalization, the difference of RCS between these two points is reduced. In other words, azimuth helps to enhance the RCS of points close to zero radian. Since the azimuths between −π/3 and π/3 are all approximately less than 1 radian, the effect of shrinkage has a large scope. A radar image constructed from RCS and a radar image constructed from azimuth-weighted RCS are compared in Figure 5, where the green dashed rectangles are indicative of a point with enhanced RCS and the blue dashed rectangles represent a point with a similar azimuth.

In summation, the extended radar channel is constructed as follows:

(14) $$E_{CRFRD}^{radar}=\left\{E_{CRF-Net}^{radar},v_{x},v_{y},\sigma_{\theta }\right\},$$

where $E_{CRF-Net}^{radar}=\{d,\sigma \}$.

### 3.3. Dual-CBAM-FPN

**Figure 6:**
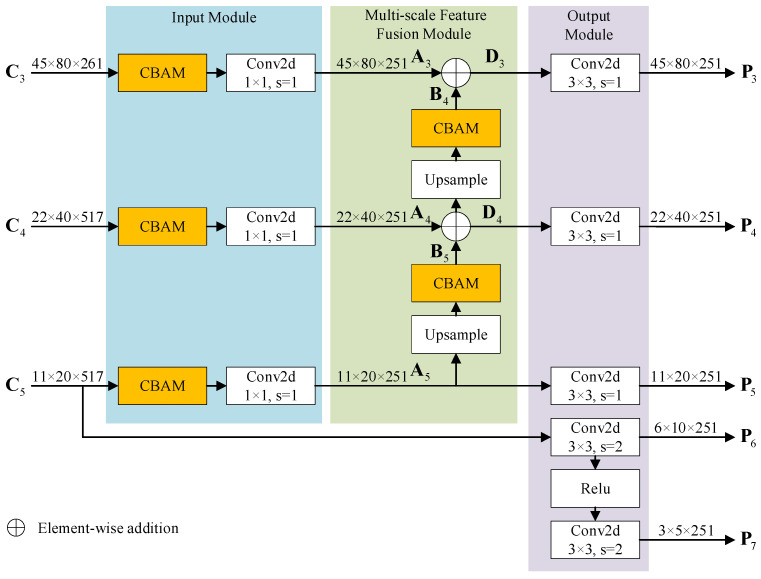
DCF is composed of the input module, the multi-scale feature fusion module, and the output module. The input module consists of CBAM and 2D convolution with 1 × 1 convolution kernel and stride 1. The multi-scale feature fusion module is a bottom–up network in which the feature is upsampled, processed by CBAM, and added to the output of the input module. The output module achieves the multi-scale features from the multi-scale feature fusion module as well as from the input module using 2D convolution and relu operation.

The structure of the dual-CBAM-FPN module is depicted in Figure 6, which comprises the input module, the multi-scale feature fusion module, and the output module.

The input module consists of CBAM, as described in Section 2.4, and 2D convolution with a 1 × 1 convolution kernel and stride 1. $C_{3}$, $C_{4}$, and $C_{5}$ are the three high-level features extracted by the feature extraction and fusion module of the CRFRD. $C_{4}$ and $C_{5}$ have 517 channels, 512 of which are extracted by VGG16, while the remaining 5 are obtained from a radar image. Meanwhile, $C_{3}$ possesses 261 channels, with 256 channels extracted by VGG16 and 5 that are obtained from the radar image. The 2D convolution reduces the channel dimensions of $C_{i}$ to match the number of subsequent channels. The weighted features $A_{3}$, $A_{4}$, and $A_{5}$ are obtained after the processing of the input module,

(15) $$A_{i}={\mathrm{Conv}}_{1\times 1}\left(M\left(C_{i}\right)\right),i=3,4,5,$$

where M(⋅) represents the CBAM processing and ${\mathrm{Conv}}_{1\times 1}\left(\cdot \right)$ represents 2D convolution with 1 × 1 convolution kernel.

The multi-scale feature fusion module is a bottom–up network that begins with $A_{5}$. As described by Equation (2), the feature is upsampled and processed via CBAM to obtain $B_{i}$, which is then added to $A_{i-1}$ to obtain $D_{i-1}$.

(16) $$B_{i}=M\left(\mathrm{Upsample}\left(A_{i}\right)\right),\;i=4,5,$$

(17) $$D_{i-1}=A_{i-1}+B_{i}\;i=4,5.$$

For the output module, $D_{i}$ is processed by 2D convolution with 3 × 3 convolution kernel and stride 1 to obtain $P_{i}$. Furthermore, $P_{5}$ is obtained from $A_{5}$, $P_{6}$ is obtained from $C_{5}$, and $P_{7}$ is obtained from $P_{6}$. Since it is necessary to carry out further concatenation with the five channels of the radar image, the number of channels of $P_{i}$ must be set to 251 to maintain the number of channels after fusion at 256. The final output of the dual-CBAM-FPN module is represented by the multi-scale features $P_{3},P_{4},P_{5},P_{6},P_{7}$.

(18) $$P_{i}=\{\begin{array}{c} {\mathrm{Conv}}_{3\times 3}\left(D_{i}\right),\\ {\;\mathrm{Conv}}_{3\times 3}\left(A_{i}\right),\;\\ {\mathrm{Conv}}_{3\times 3}\left(C_{i-1}\right),\\ {\mathrm{Conv}}_{3\times 3}\left(\mathrm{Relu}\left(P_{i-1}\right)\right), \end{array}\begin{array}{c} i=3,4\\ i=5\\ i=6\\ i=7 \end{array},$$

where ${\mathrm{Conv}}_{3\times 3}\left(\cdot \right)$ represents 2D convolution with 3 × 3 convolution kernel used to alleviate the aliasing effect of upsampling.

The CBAM in the input module serves to refine feature representations of the input, emphasizing the interdependencies between channels and spatial regions. The CBAM in the multi-scale feature fusion module highlights features for specific scales. By inserting CBAM into both the input and the multi-scale feature fusion of FPN, the high-level and low-level features can be more effectively fused, which is beneficial for the detection of small or occluded objects.

## 4. Experiment and Analysis of Results

In this section, we evaluate our proposed CRFRD on the NuScenes dataset and real data. We begin by introducing the dataset, along with a detailed description of the experimental settings and evaluation criteria. After that, the numerical results and the visualization results of comparative experiments are presented. Finally, to validate the effectiveness of our proposed modules, several ablation studies are conducted.

### 4.1. Dataset and Experimental Settings

As we are unable to access open datasets for RSUs of mmWave radar sensors and camera sensors, in our experiments, we train and evaluate our model on the simplified NuScenes dataset to mimic the scenario of RSUs and then test it on the real data collected in the RSU scenario.

NuScenes is a comprehensive sensor dataset comprising data from six cameras and five mmWave radars [38]. However, we only employ the front camera and the front radar, as was the case in a previous study with a similar objective [19]. The original resolution of the camera images in the NuScenes dataset was 1600 × 900; however, we reduce this to 640 × 360, which allows the training to be completed more quickly. The front radar samples and the front camera samples in the NuScenes dataset are processed by the NuScenes generator module, providing a total of 20,480 training samples, 6839 validation samples, and 6830 test samples. We then classify the obtained samples based on what they depict; the seven chosen categories are human, bicycle, bus, car, motorcycle, trailer, or truck.

**Figure 7:**
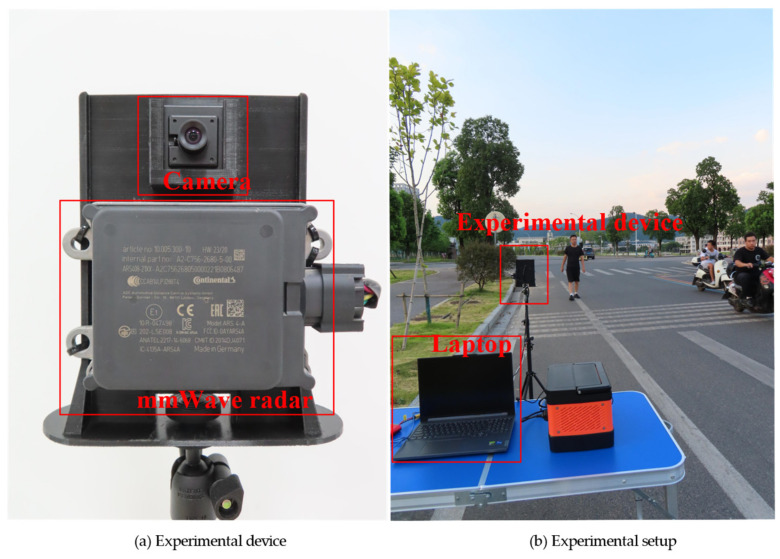
Experimental device and experimental setup: (**a**) is the experimental device composed of the mmWave radar and the camera; (**b**) is the experimental setup. The experimental device is raised up to 1.0 m high, and all the data are transferred to a laptop for storage.

To verify the proposed model’s performance in RSUs scenarios, an experiment is conducted on the roadside. We use a ARS408-21 mmWave radar manufactured by German Continental to obtain the radar point clouds and a USB camera to capture video footage. The device used in the experiment is depicted in Figure 7a. The parameters of ARS408-21 are as follows: the cycle time is approximately 72 ms; the maximum distance is 70 m for near range and 250 m for far range; the distance resolution is 0.39 m for near range and 1.79 m for far range; the velocity range is −400 km/h to 200 km/h; and the velocity resolution is 0.1 km/h. The camera is configured with an output image revolution of 640 × 480 and a frame rate of 20 fps. The experimental device is raised up to 1.0 m high and the data are transferred to a laptop for storage. The experimental setup is shown in Figure 7b. Data are collected from three scenes: scene 1, which is recorded during the daytime; scene 2, which depicts cloudy conditions; and scene 3, which takes place at night. To address the sparsity of the radar point cloud, we accumulate the point cloud data from 3 radar scans as a single frame, which is approximately 0.25 s. The data collection duration is approximately 9.5 s per scene, and in this time, 39 radar point clouds data are obtained. The corresponding 39 images are selected via time calibration, where the radar data are matched to the nearest camera data. The final test set comprises 117 samples.

We train, evaluate, and test the model on a device with 128 GB RAM and GPU configured as an RTX 3090 and CUDA 11.3. The most appropriate learning rate contributes to the model’s strong convergence. The initial learning rate is set to 2 × 10^−5^. Similar to the baseline CRF-Net [17], we conduct training for 25 epochs with a batch size of 1, which helps to prevent model overfitting.

### 4.2. Evaluation Criteria

In this paper, the weighted mean average precision (wmAP) [17] is utilized to evaluate the performance of the object detection model. The wmAP is calculated as a weighted mean of the average precision (AP) for each category.

(19) $$\mathrm{wmAP}=\frac{1}{N}\cdot \sum_{i=1}^{C}\left(N_{i}\cdot {\mathrm{AP}}_{i}\right),$$

where N represents the total number of objects in all categories; C represents the number of object categories (and C=7 in this paper); $N_{i}$ represents the number of the i-th category of object; and $AP_{i}$ represents the average accuracy of the i-th category of object.

(20) $$\mathrm{AP}=\int_{0}^{1}p\left(r\right)dr,$$

where p(r) represents the precision-recall curve; p represents the precision; and r represents the recall. When calculating the precision and recall, the intersection over union (IoU) is set to 0.5.

### 4.3. Comparative Experiments

To verify the effectiveness of the CRFRD model, we compare its detection results with one image-only network RetinaNet [32] and five millimeter-wave radar and camera fusion models that generate 2D bounding boxes: CRF-Net, the research conducted by Nabati and Qi [39], REF-Net [40], RSA + CA2 [41], and the research conducted by H. Sun et al. [42]. We do not compare our results with those described in reference [3], in which mmWave radar data and camera data for intelligent RSUs are fused at the feature level, because the authors of that study trained and evaluated the model’s performance based on their own dataset. The comparison results are summarized in Table 1, describing the AP for each category as well as the wmAP. The best results are written in bold text, while the second-best results are underlined. The results of CRF-Net are reproduced in the table below, and the wmAP is 43.89%, which is consistent with the reported result of 43.95%. The results of other models are directly cited from the referenced papers. To further compare the computational complexity of our method and the CRF-Net, we also illustrate two metrics, parameters [10] and frames per second (FPS) [43], which are usually used to evaluate the computational burden of deep learning models. The number of parameters indicates the memory occupied, while frames per second (FPS) is indicative of the inference time.

As illustrated in Table 1, according to wmAP, the CRFRD outperforms all the other models, especially for the image-only model RetinaNet. The wmAP of CRFRD surpasses that of RetinaNet, CRF-Net, Nabati and Qi, REF-Net, RSA + CA2, and H. Sun et al. by 1.45%, 1.14%, 0.54%, 0.27%, 1.11%, and 0.15%, respectively. The CRFRD achieves the best AP performance in two categories, human and car, and achieves the second-best performance in three categories, bicycle, motorcycle, and trailer; as such, it can be considered the best model in Table 1. These outcomes demonstrate the effectiveness of the proposed CRFRD model in improving detection performance.

Moreover, Table 1 shows that the computational complexity of the CRFRD has a slight depravation compared to the CRF-Net, where the parameters of CRFRD are 2.6% greater than those of CRF-Net, and the FPS of CRFRD is 3.6% less than that of CRF-Net. The performance of the proposed CRFRD is improved by sacrificing the computational complexity. Furthermore, as the radar data preprocessing also takes time, we have included the preprocessing times of radar data for one image of CRF-Net and CRFRD here; these were 0.15 milliseconds and 0.18 milliseconds, respectively.

**Figure 8:**
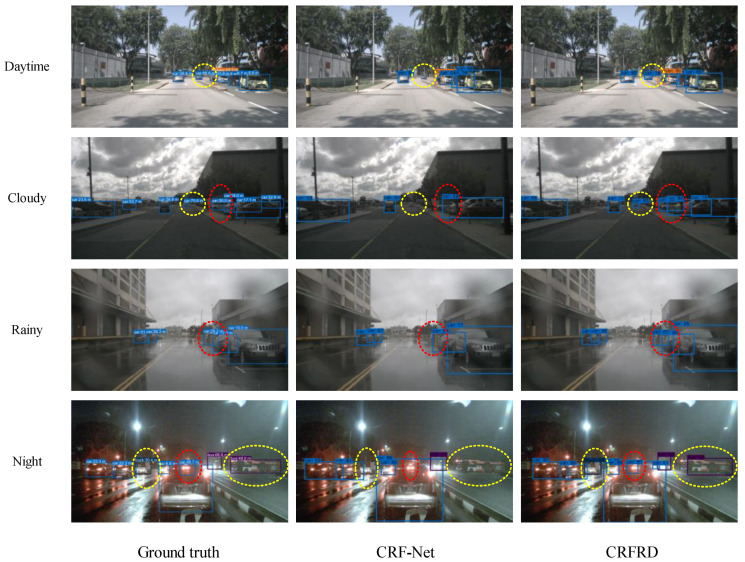
Visualization results of CRF-Net and CRFRD. Each row shows the examples of detection result in a distinct scene during the daytime, cloudy conditions, rainy conditions, and night, respectively. The images in the first column show the ground truth of the object in each scene. The second column contains the detection results of CRF-Net, and the third column contains the detection results of CRFRD. The numbers in the ground-truth images represent the distance between the target and the detection platform. The numbers in the detection results of CRF-Net and CRFRD represent the probability that the target will fit into this category. The yellow dashed ellipses indicate the small objects that are missed by CRF-Net but successfully detected by CRFRD, and the red dashed ellipses point out the occluded objects that are missed by CRF-Net but successfully detected by CRFRD.

Providing further evidence of how the CRFRD enhances the detection performance, several visualizations provided by CRF-Net and CRFRD are depicted in Figure 8. Each row in Figure 8 exemplifies a detection result from a distinct scene, during the daytime, in cloudy conditions, in rainy conditions, and at night, respectively. The cloudy, rainy, and night-time scenes pose difficulties for camera detection, which supports the advantages of millimeter-wave radar and camera fusion. The images in the first column show the ground truth of the object in each scene. The second column contains the detection result of CRF-Net, while the third column depicts the detection result of CRFRD. The yellow dashed ellipses and the red dashed ellipses indicate the small and the occluded objects that are overlooked by CRF-Net but successfully detected by CRFRD.

For each scene, several small objects and occluded objects are overlooked by CRF-Net but successfully detected by CRFRD. Specifically, in the daytime scenario, there is a car at a distance of 88.8 m that goes undetected by CRF-Net but is correctly identified by CRFRD. In the cloudy scenario, there is a car at a distance of 70.6 m that is overlooked by CRF-Net but recognized by CRFRD, and a car at a distance of 30.0 m that is occluded by another car is missed by CRF-Net but is successfully identified by CRFRD. In the rainy scenario, there is a car at a distance of 28.2 m that is occluded by another car and is not detected by CRF-Net but is identified by CRFRD. In the night-time scenario, there is a truck at a distance of 30.4 m that is not observed by CRF-Net but is recognized by CRFRD, and there is a bus with a distance of 48.0 m that is not detected by CRF-Net but is successfully detected by CRFRD. Moreover, there is a car with a distance of 48.7 m that is occluded by another car that is overlooked by CRF-Net but is identified by CRFRD. These results demonstrate CRFRD’s effective detection of both small and occluded objects, which is essential for improving the amount of time intelligent RSUs take to identify a hazard.

**Figure 9:**
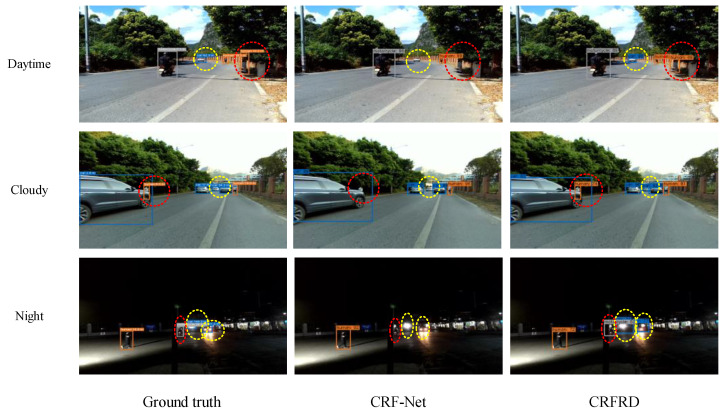
Examples of the visualization results of CRF-Net and CRFRD using real data. Each row depicts the detection results for a distinct scene recorded during the daytime, in cloudy conditions, or at night, respectively. The images in the first column show the ground truth of the object in each scene. The second column depicts the detection results of CRF-Net, while the third column showcases the detection results of CRFRD. The numbers in the ground-truth images represent the distance between the target and the detection platform. The numbers in the detection results of CRF-Net and CRFRD represent the probability that the target falls within a specific category. The yellow dashed ellipses highlight the small objects that are overlooked by CRF-Net but successfully detected by CRFRD, and the red dashed ellipses indicate the occluded objects that are missed by CRF-Net but correctly identified by CRFRD.

To test the performance of CRFRD in the RSU scenario, we evaluate CRF-Net and CRFRD using real data and illustrate some examples of the visualization results. Since the number of samples in the test set is limited, it is difficult to evaluate the AP for each category. Therefore, in the test experiment involving real data, we count the number of targets that are detected correctly for each frame to evaluate the performance of the models. As we annotate the ground truth of the targets, which include the bounding boxes and categories, a correctly detected target indicates that the IoU of the predicted bounding box and the true bounding box is larger than 0.5 and the predicted category is correct. Examples of the visualization results of CRF-Net and CRFRD using real data are shown in Figure 9. Each row depicts the detection result for a specific scene recorded during the daytime, in cloudy conditions, and at night, respectively. The images in the first column show the ground truth of the object in each scene. The second column showcases the detection results of CRF-Net, while the third column depicts the detection results of CRFRD. The numbers in the ground-truth images represent the distance between the target and the detection platform. The numbers in the detection results of the CRF-Net and CRFRD represent the probability that the target is in a specific category. The yellow dashed ellipses highlight the small objects that are overlooked by CRF-Net but successfully detected by CRFRD and the red dashed ellipses indicate the occluded objects that are missed by CRF-Net but successfully identified by CRFRD.

Figure 9 shows that in the image of the daytime scenario, there are four targets, of which two are correctly detected by CRF-Net, while all four are successfully identified by the proposed CRFRD. CRF-Net misidentifies a car at a distance of 62.3 m and an occluded human, but CRFRD does not exhibit the same limitations. In the image of the cloudy scenario, there are six targets, four of which are correctly detected by CRF-Net, while all six targets are correctly identified by the proposed CRFRD. CRF-Net misidentifies a car at a distance of 34.3 m and an occluded human, while CRFRD successfully identifies them. In the image of the night-time scenario, there are four targets, of which one is correctly detected by CRF-Net, while all four are successfully identified by the proposed CRFRD. CRF-Net fails to detect a car at a distance of 144.3 m, a car at a distance of 51.2 m, and an occluded motorcycle, while CRFRD successfully identifies them. The superiority of the model in detecting small targets and occluded targets is verified by the data collected from real roadside scenes.

By gathering the statistics of all 39 images, the results of each scene can be evaluated. In the daytime scenes, there are 142 targets; of these, 50 targets are correctly identified by CRF-Net, while 74 targets are correctly identified by the proposed CRFRD. In the scene of cloudy conditions, there are 216 targets, of which 150 are successfully identified by CRF-Net, while 201 targets are correctly identified by the proposed CRFRD. In the scenes recorded during the night, there are 128 targets, of which 36 targets are detected correctly by CRF-Net and 67 targets are correctly identified by the proposed CRFRD. The evaluation results verify the superior performance of the CRFRD in various scenes.

### 4.4. Ablation Study

To verify the effectiveness of the RCE and DCF modules in CRFRD and the influence of θ and $\sigma_{\theta }$ in the RCE module on enhancing the detection performance, several ablation studies are performed.

Table 2 depicts the results of the ablation study of two main modules: RCE and DCF. As shown, the wmAP decreases when any module is removed. When both modules are utilized, the model achieves the highest wmAP of 45.03%, performs best on AP for three categories, and performs the second best on AP for two categories, demonstrating a superior performance compared to any other case in which one or more modules are removed. When RCE module is removed, the radar input is reduced to two channels where the features are not rich enough. When DCF module is removed, during the process of feature extraction and fusion, the feature map has no attention mechanism with which to extract key information and is unable to make full use of the information between channels and spaces, which affects its ability to detect small and occluded objects. Consequently, both the RCE and the DCF modules are indispensable in CRFRD.

Table 3 shows the results of the ablation study about azimuth θ and azimuth-weighted RCS $\sigma_{\theta }$ in the RCE module as additional radar-extended channels along with velocity. The observations reveal that using azimuth-weighted RCS $\sigma_{\theta }$ as a parameter to construct additional radar channels offers the highest wmAP of 44.74%. Specifically, it performs best on AP for three categories and performs second best for two categories, surpassing any other scenarios. These results verify that the utilization of richer redundant information $\sigma_{\theta }$ improves the detection performance more effectively compared to redundant information θ.

## 5. Conclusions

This paper proposes a camera-radar fusion model named CRFRD, which incorporates an RCE module and a DCF module into the CRF-Net model to enhance object detection performance. By adding the RCE and DCF modules simultaneously, we enrich the radar feature representation and direct the model’s focus toward the important information. In the RCE module, we design an azimuth-weighted RCS parameter that leverages the secondary redundant information to generate richer feature representation and extend three radar channels, namely, the velocity in the x-direction, the velocity in the y-direction, and azimuth-weighted RCS. In the DCF module, the dual-CBAM-FPN structure is proposed, enabling the model to focus on important feature information by inserting CBAM at the input and the fusion process of FPN simultaneously. This is beneficial for the detection of small or occluded objects, since the high-level and low-level features are more effectively fused. Comparative experiments conducted on the NuScenes dataset and real data demonstrate the superior performance of the CRFRD model compared to CRF-Net, particularly with regard to the detection of small and occluded objects, and the wmAP increases from 43.89% to 45.03%. Additionally, our ablation studies verify the indispensability of the RCE and DCF modules and the effectiveness of azimuth-weighted RCS.

Several of the obstacles observed in this study warrant further exploration in the future; for example, the proposed CRFRD method is more likely to overlook or misidentify motorcycles, buses, and trailers. The reason for this may be that azimuth-weighted RCS only enhances the RCS of the targets whose azimuth is close to zero radian; for targets whose azimuth is far beyond zero radian, azimuth-weighted RCS plays a limited role. Therefore, in future work, we will seek more suitable feature representation for targets that have a large azimuth.

## Figures and Tables

**Table 1:** The result of comparative experiments. The best results are written in bold text, while the second-best results are underlined.

	Class	Human	Bicycle	Bus	Car	Motorcycle	Trailer	Truck	wmAP	Parameters	FPS
Model											
RetinaNet [32]		40.25	7.14	17.19	53.33	19.36	5.95	25.00	43.58		
CRF-Net		38.41	14.99	33.67	52.96	**26.60**	**18.37**	25.85	43.89 (43.95 ^⊕^)	22.45 M	4.12
Nabati and Qi [39] *		27.59	**25.00**	**48.30**	52.31	25.97		**34.45**	44.49		
REF-Net [40]									44.76		
RSA + CA2 [41]		38.87	15.19	35.39	52.42	24.09	15.02	28.53	43.92		
H. Sun et al. [42]									44.88		
CRFRD		**40.35**	17.69	33.08	**54.27**	26.22	17.73	27.46	**45.03**	23.12 M	3.97

* The results in this row were reported by Nabati and Qi [39]. ^⊕^ The results were reported by CRF-Net [17].

**Table 2:** Ablation study of two main modules. The √ means that this module is used in the model. The best results are written in bold text, while the second-best results are underlined.

RCE	DCF	Human	Bicycle	Bus	Car	Motorcycle	Trailer	Truck	wmAP
		38.41	14.99	33.67	52.96	26.60	**18.37**	25.85	43.89
√		39.66	15.23	**34.76**	53.79	**27.69**	16.29	26.43	44.74
	√	40.26	14.78	27.25	53.14	26.35	17.37	**27.48**	44.41
√	√	**40.35**	**17.69**	33.08	**54.27**	26.22	17.73	27.46	**45.03**

**Table 3:** Ablation study of θ and $\sigma_{\theta }$ as additional channel/channels in the RCE module. The √ means that this parameter is used in the model. The best results are written in bold text, while the second-best results are underlined.

θ	$\sigma_{\theta }$	Human	Bicycle	Bus	Car	Motorcycle	Trailer	Truck	wmAP
		39.07	13.20	28.63	53.40	22.91	**18.21**	26.82	44.20
√		39.64	14.44	31.41	52.93	**28.50**	17.05	**29.18**	44.49
	√	39.66	**15.23**	**34.76**	**53.79**	27.69	16.29	26.43	**44.74**
√	√	**39.90**	13.28	30.20	53.68	20.40	16.86	26.34	44.52

## Data Availability

The raw data supporting the conclusions of this article will be made available by the authors on request.
